# Infection with *Toxoplasma gondii* does not Alter TNFα and IL-6 Secretion by A human Astrocytoma Cell Line

**DOI:** 10.1155/S0962935194000402

**Published:** 1994

**Authors:** H. Pelloux, J. Ricard, M.-F. Nissou, J.-C. Renversez, J.-P. Vuillez, A. Meunier, P. Ambroise-Thomas

**Affiliations:** 1 Dédpartement de Parasitologie-Mycologie Médicale et Moléculaire CNRS ERS 15, Faculté de Médecine Université Joseph Fourier Grenoble 1, Grenoble Cedex 38043 France; 2 Laboratoire de Neuro-Biophysique INSERM U 318 Faculté de Médecine Université Joseph Fourier Grenoble 1, Grenoble Cedex 38043 France; 3 Laboratoire de Biochimie A Faculté de Médecine Université Joseph Fourier Grenoble 1, Grenoble Cedex 38043 France; 4 Département de Biophysique CNRS URA 1287 Faculté de Médecine Université Joseph Fourier Grenoble 1, Grenoble Cedex 38043 France

## Abstract

The secretion of tumour necrosis factor-α (TNFα), interleukin-1α (IL-α) and interleukin-6 (IL-6) by a human astrocytoma cell fine was studied 1 h, 3 h, 6 h and 24 h after infection with tachyzoites from three *Toxoplasma gondii* strains (virulent, RH; cystogentc, 76K and Prugniaud strains). The astrocytoma cell fine constitutively secreted TNFα and IL-6, but no IL-1α. A positive control was obtained by stimulation with phorbol esters inducing a significant increase (p < 0.05) in TNFα and IL- 6 secretion but not in IL-1α, while lipopolysaccharide (alone and after priming), interferon gamma, ionophore A 23187 and sera positive to *T. gondii* did not induce any increase in cytokine levels. None of the tachyzoites, whatever their virulence, induced a significant increase in cytokine production at any time in the study. Tachyzoites did not inhibit the secretion induced by phorbol esters.


Mediators of Inflammation 3, 291–295 (1994)

### Infection with *Toxoplasma gondii* does not alter TNFα and IL-6 secretion by a human astrocytoma cell line

H. Pelloux,[1,CA] J. Ricard,[1] M.-F. Nissou,[2] J.-C. Renversez,[3] J.-P. Vuillez,[4] A. Meunier[1] and P. Ambroise-Thomas[1]

[1]Département de Parasitologie-Mycologie Médicale et Moléculaire, CNRS ERS 15,[2]Laboratoire de Neuro-Biophysique INSERM U 318, [3]Laboratoire de Biochimie A and [4]Département de Biophysique CNRS URA 1287, Faculté de Médecine, Université Joseph Fourier, Grenoble 1, 38043 Grenoble Cedex, France

[CA] Corresponding Author

**The secretion of tumour necrosis factor-α (TNFα), interleukin-1α (IL-1α) and interleukin-6 (IL-6) by a human astrocytoma cell line was studied 1 h, 3 h, 6 h and 24 h after infection with tachyzoites from three *Toxoplasma gondii* strains (virulent, RH; cystogenic, 76K and Prugniaud strains). The astrocytoma cell line constitutively secreted TNFα and IL-6, but no IL-1α. A positive control was obtained by stimulation with phorbol esters inducing a significant increase ($p < 0.05$) in TNFα and IL-6 secretion but not in IL-1α, while lipopolysaccharide (alone and after priming), interferon gamma, ionophore A 23187 and sera positive to *T. gondii* did not induce any increase in cytokine levels. None of the tachyzoites, whatever their virulence, induced a significant increase in cytokine production at any time in the study. Tachyzoites did not inhibit the secretion induced by phorbol esters.**

**Key words:** Astrocytoma cells, Cytokine, *Toxoplasma gondii*

### Introduction

*Toxoplasma gondii* is a worldwide intracellular obligate parasite, which causes severe and frequent lesions (mainly cerebral) in AIDS patients.[1] The pathophysiology of reactivation of toxoplasmosis in immunocompromised patients is still not clear, and the exact mechanisms of cyst rupture, including the role of cytokines remain unknown.[2,3] While interferon-γ (INFγ) is involved in protective immunity,[4] its precise role and mode of action is not known. Tumour necrosis factor-α (TNFα), a cytokine which is involved in infectious and inflammatory diseases, has not been extensively studied in toxoplasmosis. Its role during toxoplasmic infection in mice is controversial,[5,6] and TNFα would seem to be implicated in human and animal toxoplasmosis.[7–9] Interleukin-6 (IL-6) and interleukin-1α (IL-1α) play a role in parasitic diseases[10] and in mouse toxoplasmosis.[11] Their transcripts have been detected in brains of mice infected with *T. gondii*.[12] Thus, these three cytokines (TNFα, IL-6, IL-1α) may be involved in the development of immunity that occurs in immunocompetent hosts after infection with *T. gondii*, as they are in the viral infections (particularly HIV).[13]

Cerebral parasitic multiplication following cyst reactivation is the main event of toxoplasmic disease in patients with Acquired Immuno Deficiency Syndrome, but to our knowledge, few publications have studied the parasitic multiplication, the cyst formation and rupture in astrocytes, which are cerebral host cells for *T. gondii*.[4] Moreover, some authors have recently suggested that differences between *T. gondii* strains could be the cause of virulence differences in animals and perhaps in man, in association with the host immunological variations.[14–17] Thus, the aim of our work was to study the secretion of TNFα, IL-1α and IL-6 by a human astrocytoma cell line infected by three *T. gondii* strains with different degrees of virulence.

### Materials and Methods

*Astrocytoma cell line:* The GHE (Glioblastome Humain E) cell line used for this study was derived from a surgical specimen of a primary brain tumour (grade II astrocytoma according to the classification of Kernohan and WHO). Briefly, the tumour specimen was minced and dissociated into single cells following incubation for 10 min in 0.5% trypsin in DMEM medium (Sigma, St Louis, MO).[18] The cell line was then routinely grown in 25 $cm^2$ tissue culture flasks in DMEM medium supplemented with 10% foetal calf serum (DAP, Vogelgrun, France) and antibiotics (100 U penicillin and 50 μg streptomycin per ml). Culture was maintained at 37°C in humidified air containing 5% $CO_2$, and cells were subcultured when confluent.

After establishment, growth curves and cell doubling times were determined. The morphological

© 1994 Rapid Communications of Oxford Ltd features of the cell line were recorded with a phase-contrast microscope and camera. The glial origin of the cells was established by the presence of the glial fibrillary acidic protein (GFAP) and the S100 protein, markers for astrocytic differentiation, and with morphological histological criteria as described elsewhere.[18]

The absence of *Mycoplasma* contamination was checked using the *Mycoplasma* detection kit from Boehringer (Meylan, France).

*Toxoplasma gondii strains:* Three *T. gondii* strains were used: the virulent RH, and the chronic 76K and Prugniaud strains.[15,19] The RH strain never forms cysts in mice, and infection kills mice after 3 or 4 days. In contrast, mice infected with the 76K and the Prugniaud strains do not die and cysts are present in mouse brains. The RH strain was obtained by *in vitro* culture in human fibroblast MRC5 cells (bioMérieux, Marcy l'Etoile, France). The RH tachyzoites were collected from the culture supernatant (1 200 × **g** for 10 min) after 4 days and then counted in a Neubauer cell. The Prugniaud and 76K tachyzoites were obtained after inoculation of Swiss mice (OF1 strain, Iffa Credo, L'Arbresle, France) treated with corticosteroids. Briefly, mice received 1 mg i.m. of hydrocortisone acetate (Roussel, Paris, France) for 5 days. Tachyzoites were injected intraperitoneally, and cortisone treatment was prolonged for 10 days. After tachyzoite multiplication, the peritoneal fluid was harvested, the tachyzoites were washed (1 200 × **g** for 10 min) and counted. Their viability was assayed by the ethidium bromide–acridine orange assay (Becton Dickinson, Oxnard, CA), and only parasite preparations with a viability > 95% were used.

*TNFα assay:* The immunoradiometric assay kit for detection of human TNFα (Medgenix, Brussels, Belgium) was used. The medium containing astrocytes or parasites was free of TNFα (< 6 pg/ml).

*IL-1α and IL-6 assay:* The techniques used were the competitive sandwich immuno enzymatic methods (Immunotech, Marseille, France) for IL-6 and IL-1α interleukins. The medium containing astrocytes and parasites was free of these cytokines (< 20 pg/ml).

*Study of cytokine secretions:* The GHE cells were distributed into 24-well plates at $1.5 \times 10^4$ cells per well in 1 ml of culture medium and then left to multiply for 1 week at 37°C, 5% $CO_2$. Experiments were performed when the cell monolayers were confluent (approximately $1.5 \times 10^5$ cells per well). Fresh medium was added to the cells at T0.

*T. gondii* tachyzoites of the different strains ($1.5 \times 10^5$ per well) were added to the wells at T0. The ratio cells : parasites was then of approximately 1:1. In some experiments, $10^6$ parasites were added to the wells.

Depending on the experiments, lipopolysaccharide (LPS) (10 or 1 μg/ml; Calbiochem, Meudon, France), positive sera, human recombinant interferon-γ (INFγ) (100 U/ml); Boehringer, Meylan, France), phorbol esters (phorbol-12-myristate-13-acetate (PMA) $10^{-8}$ M; Sigma, St Louis, MO), ionophore A 23187 ($10^{-6}$ M; Sigma), or polymyxin B (1 μg/ml; Pfizer, Orsay, France) in order to inhibit LPS action, were added to the wells at T0.

Supernatants were collected from corresponding wells at different times (1 h, T1; 3 h, T2; 6 h, T3; 24 h, T4). For some experiments, parasites were allowed to grow in cells for 2 h before stimulation.

*Immunofluorescence staining:* The presence of parasites in GHE cells was revealed by indirect immunofluorescence staining. The cell monolayer was fixed and then dried. A rabbit polyclonal anti-*Toxoplasma* antibody was added and incubated for 30 min at 37°C. After washing, a fluorescein-labelled anti-rabbit IgG antibody (Institut Pasteur, France) was used to reveal the presence of parasites with an UV light microscope.

*Positive sera:* Twelve sera were collected from twelve patients with high levels of IgG antibodies to *T. gondii* (640 to 1280 IU/ml, indirect fluorescent antibody technique).

*Statistical analysis:* A statistical analysis was performed using analysis of variance and Kolmogorov–Smirnov tests.

### Results

*Infection of GHE cells:* The percentage of cells infected by *T. gondii* (assessed by immunofluorescence staining) was 10% after 1 h and 25% after 24 h when $10^5$ parasites of the RH strain were added to the cells. When $10^6$ parasites were added, those percentages were 20% and 45% respectively. Those infection rates were not significantly different with the parasites from the chronic strain, but the average numbers of parasites per infected cell were higher for the virulent strain—approximately 5 parasites/cell for the RH strain after 24 h, and less than 2 parasites/cell for 76K. These infection rates were not significantly modified by INFγ (100 U/ml).

The microscopic examination showed areas of lysed cells in the case of infection by the RH strain, which were not present in the case of infection with chronic strains.

*Secretions without stimulation (negative control):* In our experimental conditions, the astrocytoma cell line constitutively secreted low amounts of TNFα (Fig. 1), higher amounts of IL-6 (Fig. 2) and no IL-1α. The secretions of TNFα and IL-6 increased with time of sampling (the medium was changed at T0). No decrease in secretion was observed when polymyxin

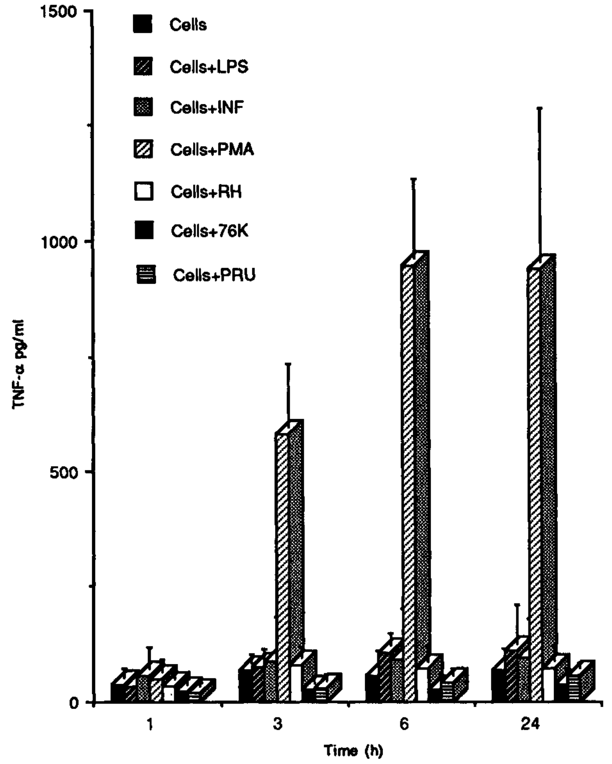

FIG. 1. TNF produced by astrocytoma cells. The values for TNFα represent the mean of nine experiments with astrocytoma cells alone (control), five experiments with cells plus LPS (10 μg/ml) or INFγ (100 U/ml), four experiments with cells plus PMA ($10^{-8}$ M), four experiments with cells plus RH *T. gondii* strain, two experiments with cells plus 76K strain and three experiments with cells plus Prugniaud strain. The supernatants were collected 1 h, 3 h, 6 h and 24 h after stimulation. The same results were obtained with LPS at 1 μg/ml. The results with astrocytoma cells alone were not modified by addition of polymyxin B (1 μg/ml). Error bars represent one standard deviation.

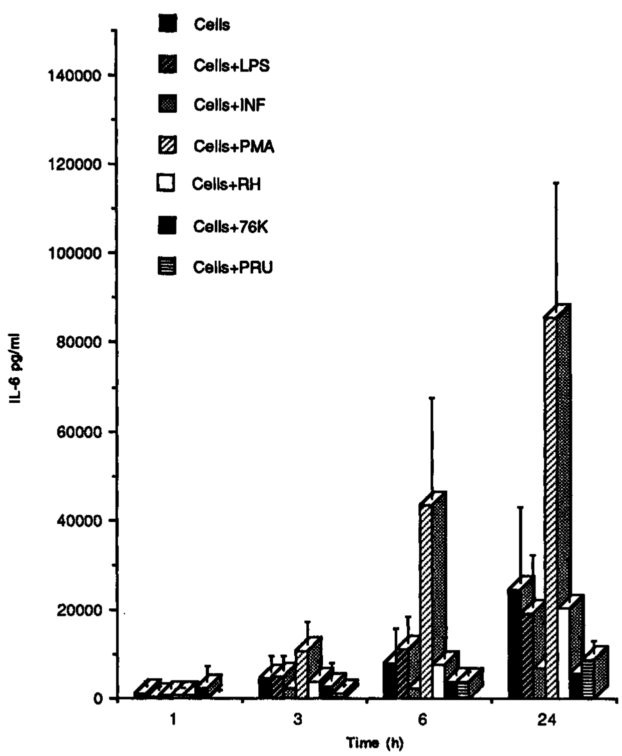

FIG. 2. IL-6 produced by astrocytoma cells. Details are the same as given in the legend to Fig. 1 except that IL-6 was used instead of TNFα.

B was added. After 48 h, the cytokine levels were not different from those noted after 24 h (data not shown).

*Positive controls:* Cells from the GHE cell line were able to secrete significantly ($p < 0.05$) higher amounts of TNFα and IL-6 at T2, T3 and T4 when stimulated by PMA than after each of the other stimulations (Figs 1 and 2). In this case the viability of the cells remained high (> 95%). DMSO (dimethylsulfoxide; Sigma) alone, the diluent of the PMA, did not induce any secretion.

Moreover, LPS alone (10 and 1 μg/ml), INFγ alone (100 U/ml), ionophore alone ($10^{-6}$ M), INFγ plus LPS at the same time, or cell priming by INFγ (8 h) and then addition of LPS, did not induce a significant increase in secretion of cytokine by these cells. Different parasite numbers ($10^5$ or $10^6$ tachyzoites) did not modify the cytokine secretions. In our experiments, the cytokine (TNFα and IL-6) levels were significantly lower ($p < 0.05$) at T1 than at T2, T3 or T4 after PMA stimulation.

*No secretion depending on the strains:* The multiplication of *T. gondii* tachyzoites did not induce the secretion of the three cytokines by the astrocytoma cells (Figs 1 and 2) whatever the strain used. The twelve IgG positive sera to *T. gondii* gave the same results.

The penetration of parasites into the cells during 2 h before the experiment did not inhibit the secretions induced by PMA (Figs 3 and 4).

### Discussion

The results show that the GHE cell line constitutively secretes TNFα and large amounts of IL-6, but no detectable IL-1α before 24 h. The constitutive secretion of TNFα is not constant in human astrocytoma cell lines: Bethea *et al.*[20] report that TNFα secretion was obtained only after stimulation in the CH235-MG cell line. The astrocytoma cells we studied do not respond to LPS and INFγ stimulation, even after priming by INFγ, contrary to the data reported by Chung and Benveniste.[21] These results are not surprising since cytokine expression or secretion by glial cells are very different depending on the cell origins and the stimulation.[22–25] We did not observe any secretion of IL-1α induced by previous TNFα secretion, as reported in a mouse model after LPS stimulation.[26] Chang *et al.*[11] showed that, in mice, the anti-*T. gondii* action of INFγ depends partly on the TNFα production induced by INFγ itself.

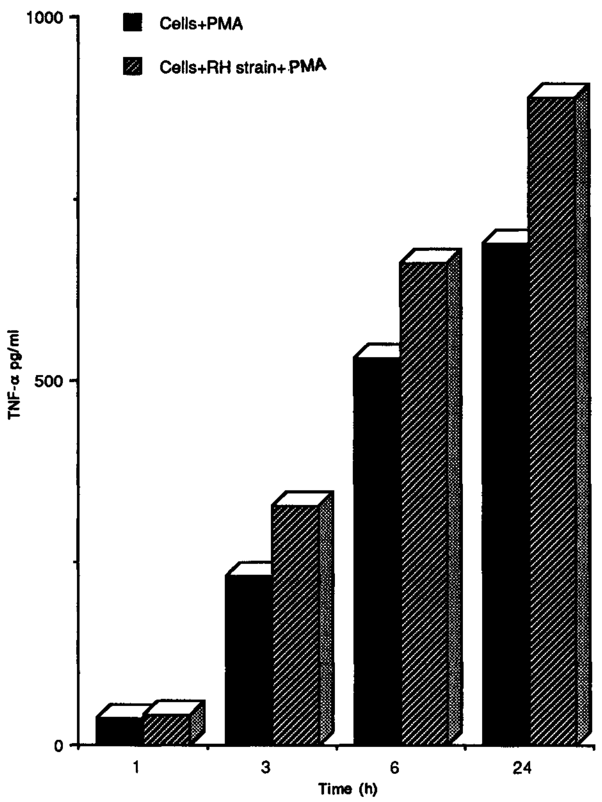

FIG. 3. TNF produced by astrocytoma cells after infection and PMA. Data obtained from one representative experiment obtained with the RH strain. Astrocytoma cells were infected with *T. gondii* and 2 h later PMA was added, simultaneously in the wells without parasites. Supernatants were collected 1 h, 3 h, 6 h and 24 h after PMA addition.

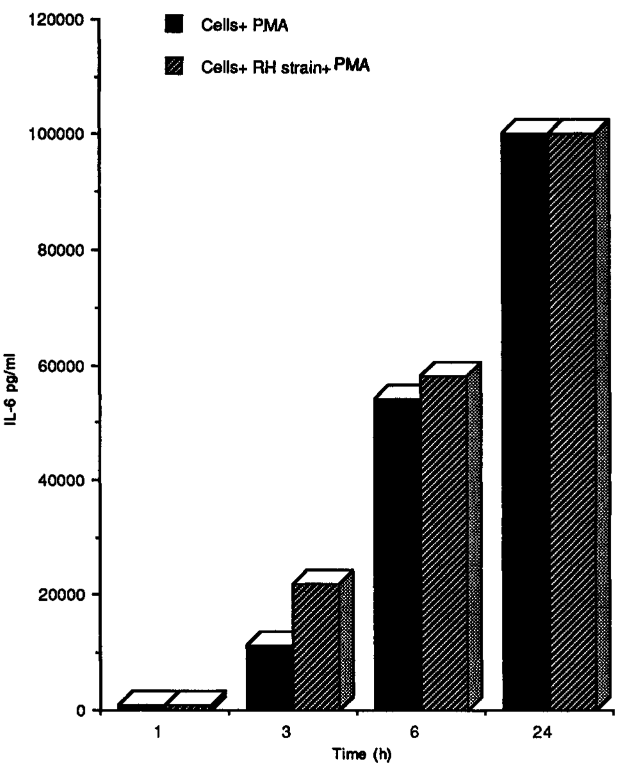

FIG. 4. IL-6 produced by astrocytoma cells after infection and PMA. Other details are the same as given in the legend to Fig. 3.

The virulent RH strain did not induce secretion of any of the three cytokines, when compared with the secretions observed with astrocytoma cells alone. Such data are consistent with our previous studies on human monocytes and macrophages concerning TNFα[8] and with the study of Friedland[27] on secretion of TNFα and IL-6 by a human monocytic cell line, but is different from the results obtained after infection of murine mononuclear cells by *Leishmania infantum* which is another obligate intracellular protozoan.[28] However, the intracellular behaviour of *T. gondii* and *L. infantum* is not the same, especially concerning the mechanisms used to escape intracellular killing, which could lead to differences in the induction of TNFα secretion.

The present model may mimic what happens after cyst rupture: tachyzoites from cystogenic strains enter brain cells around the cyst where they multiply. Sera with high anti-*Toxoplasma* IgG antibody titres did not induce this type of secretion, in contrast to what was shown with blood monocytes and monocyte-derived macrophages. The presence of exogenous INFγ, simultaneously with TNFα and IL-6 secreted by GHE cells did not lead to a decrease in infection rates. Our results are similar to those from Chao *et al.*[29] and Peterson *et al.*[30] comparing microglial cells and astrocytes in a murine model. Thus, the protective role of cerebral endogenous, astrocyte-secreted, TNFα is still unclear in man, even though it has been demonstrated to be an important factor of resistance to *T. gondii* infection in mice.[6,31]

The causes of the different degrees of virulence between *T. gondii* strains are not understood. The genotypic and phenotypic variations between the *T. gondii* strains have been demonstrated for enzymatic activities, RFLP and pathogenicity in animals and humans.[15–17] From our results, the different strains do not induce different cytokine profiles in response to a parasitic infection. Thus the assumed differences between strains, which could explain the onset of cerebral reactivation in AIDS patients, do not seem to be mediated by astrocyte cytokine production. However, cytokines produced by other cerebral cells, such as microglial cells, or recruited inflammatory cells (lymphocytes, macrophages and/or polymorphonuclear) might have an influence on parasitic multiplication and cyst rupture. Moreover, human monocytes and macrophages may secrete TNFα in the presence of specific anti-*Toxoplasma* antibodies.[8]

In conclusion the *T. gondii* protozoan does not induce (or prevent) cytokine secretion by human astrocytoma derived cells.

### References

1. Luft B, Remington JS. Toxoplasmic encephalitis in AIDS. *Clin Inf Dis* 1992; **15:** 211–222.
2. Beaman MH, Wong SY, Remington JS. Cytokines, *Toxoplasma* and intracellular parasitism. *Immunol Rev* 1992; **127:** 97–117.
3. Sher A, Coffman RL. Regulation of immunity to parasites by T cells and T cell-derived cytokines. *Annu Rev Immunol* 1992; **10:** 385–409.
4. Jones TC, Bienz KA, Erb P. *In vitro* cultivation of *Toxoplasma gondii* cysts in astrocytes in the presence of gamma interferon. *Infect Immun* 1986; **51:** 147–156.
5. Grau GE, Tacchini-Cottier F, Piguet PF. Is TNF beneficial or deleterious in toxoplasmic encephalitis? *Parasitol Today* 1992; **8:** 322–324.
6. Langermans JA, van der Hulst MEB, Nibbering PH, van Furth R. Endogenous tumor necrosis factor alpha is required for enhanced antimicrobial activity against *Toxoplasma gondii* and *Listeria monocytogenes* in recombinant gamma interferon-treated mice. *Infect Immun* 1992; **60:** 5107–5112.
7. Freund YR, Sgarlato G, Jacob CO, Suzuki Y, Remington JS. Polymorphisms in the tumor necrosis factorα (TNF-α) gene correlate with murine resistance to development of toxoplasmic encephalitis and with levels of TNF-α mRNA in infected brain tissue. *J Exp Med* 1992; **175:** 683–688.
8. Pelloux H, Chumpitazi BFF, Santoro F, Polack B, Vuillez JP, Ambroise-Thomas P. Sera of patients with high titers of immunoglobulin G against *Toxoplasma gondii* induce secretion of tumor necrosis factor alpha by human monocytes. *Infect Immun* 1992; **60:** 2672–2676.
9. Sibley LD, Adams LB, Fukutomi Y, Krahenbuhl JL. Tumor necrosis factor-α triggers antitoxoplasmal activity of IFN-γ primed macrophages. *J Immunol* 1991; **147:** 2340–2345.
10. Chen W, Havell EA, Gigliotti F, Harmsen AG. Interleukin-6 production in a murine model of *Pneumocystis carinii* pneumonia: relation to resistance and inflammatory response. *Infect Immun* 1993; **61:** 97–102.
11. Chang HR, Grau GE, Pechere JC. Role of TNF and IL-1 in infections with *Toxoplasma gondii*. *Immunol* 1990; **69:** 33–37.
12. Hunter CA, Roberts CW, Alexander J. Kinetics of cytokine mRNA production in the brains of mice with progressive toxoplasmic encephalitis. *Eur J Immunol* 1992; **22:** 2317–2333.
13. Genis P, Jett M, Bernton EW, *et al.* Cytokines and arachidonic metabolites produced during human immunodeficiency virus (HIV)-infected macrophage–astroglia interactions: implications for the neuropathogenesis of HIV disease. *J Exp Med* 1992; **176:** 1703–1718.
14. Cristina N, Liaud MF, Santoro F, Oury B, Ambroise-Thomas P. A family of repeated DNA sequences in *Toxoplasma gondii*: cloning sequence analysis, and use in strain characterisation. *Exp Parasitol* 1991; **73:** 73–81.
15. Cristina N, Oury B, Ambroise-Thomas P, Santoro F. Restriction-fragment-length polymorphisms among *Toxoplasma gondii* strains. *Parasitol Res* 1991; **77:** 266–268.
16. Darde ML, Bouteille B, Pestre-Alexandre M. Isoenzyme analysis of 35 *Toxoplasma gondii* isolates and the biological and epidemiological implications. *J Parasitol* 1992; **78:** 786–794.
17. Sibley LD, Boothroyd JC. Virulent strains of *Toxoplasma gondii* comprise a single clonal lineage. *Nature* 1992; **359:** 82–85.
18. Nissou MF. Etablissement et caractérisation de lignées cellulaires à partir de tumeurs cérébrales humaines. PhD thesis. Université Joseph Fourier Grenoble I Grenoble. France. 1989.
19. Zenner L, Darcy F, Cesbron-Delauw MF, Capron A. Rat model of congenital toxoplasmosis: rate of transmission of three *Toxoplasma gondii* stains to fetuses and protective effect of a chronic infection. *Infect Immun* 1993; **61:** 360–363.
20. Bethea JR, Yancey-Gillespie G, Benveniste EN. Interleukin-1β induction of TNFα gene expression: involvement of protein kinase C. *J Cell Physiol* 1992; **152:** 264–273.
21. Chung IY, Benveniste EN. Tumour necrosis factor-a production by astrocytes. Induction by lipopolysaccharide, IFN-γ, and IL-1β. *J Immunol* 1990; **144:** 2999–3007.
22. Hetier E, Ayala J, Bousseau A, Denèfle P, Prochiantz A. Amoeboid microglial cells and not astrocytes synthesize TNF-α in Swiss mouse brain cell cultures. *Eur J Neurosci* 1990; **2:** 762–768.
23. Lee JC, Simon PL, Young PR. Constitutive and PMA-induced interleukin-1 production by the human astrocytoma cell line T24. *Cell Immunol* 1989; **118:** 298–311.
24. Lieberman AP, Pitha PM, Shin HS, Shin ML. Production of tumor necrosis factor and other cytokines by astrocytes stimulated with lipopolysaccharide or a neurotropic virus (interleukin 1/type I interferons/interleukin 6). *Proc Natl Acad Sci USA* 1989; **86:** 6348–6352.
25. Wesselingh SL, Gough NM, Finlay-Jones JJ, McDonald PJ. Detection of cytokine mRNA in astrocyte cultures using the polymerase chain reaction. *Lymphokine Res* 1990; **9:** 177–185.
26. Zuckerman SH, Evans GF, Butler LD Endotoxin tolerance: independent regulation of interleukin-1 and tumour necrosis factor expression. *Infect Immun* 1991; **59:** 2774–2780.
27. Friedland JS, Shattock RJ, Johnson JD, Remick DG, Holliman RE. Differential cytokine gene expression and secretion after phagocytosis by a human monocytic cell line of *Toxoplasma gondii* compared with *Mycobacterium tuberculosis*. *Clin Exp Immunol* 1993; **91:** 282–286.
28. Chiofalo MS, Delfino D, Mancuso G, La Tassa E, Mastroeni P, Ianello D. Induction of tumor necrosis factor-α by *Leishmania infantum* in murine macrophages from different inbred mice strains. *Microbiol Pathogen* 1992; **12:** 9–17.
29. Chao CC, Hu S, Gekker G, Novick WJ, Remington JS, Peterson PK. Effects of cytokines on multiplication of *Toxoplasma gondii* in microglial cells. *J Immunol* 1993; **150:** 3404–3410.
30. Peterson PK, Gekker G, Hu S, Chao CC. Intracellular survival and multiplication of *Toxoplasma gondii* in astrocytes. *J Inf Dis* 1993; **168:** 1472–1478.
31. Johnson LL. A protective role for endogenous tumor necrosis factor in *Toxoplasma gondii* infection. *Infect Immun* 1992; **60:** 1979–1983.

ACKNOWLEDGEMENTS. This work was supported by A.N.R.S. (Agence Nationale de Recherche sur le SIDA). We are indebted to M. L. Darde for kindly providing the Prugniaud strain, to C. Boudin for statistical analysis and to B. F. F. Chumpitazi for critical reading of the manuscript.

**Received 21 March 1994; accepted 12 April 1994**
